# Application of enzymes as a feed additive in aquaculture

**DOI:** 10.1007/s42995-022-00128-z

**Published:** 2022-04-19

**Authors:** Qingping Liang, Mingxue Yuan, Liping Xu, Elia Lio, Fang Zhang, Haijin Mou, Francesco Secundo

**Affiliations:** 1 College of Food Science and Engineering, Ocean University of China, Qingdao, 266003 China; 2 College of Biology and Geography, Yili Normal University, Yining, 835000 China; 3 Istituto di Scienze e Tecnologie Chimiche “Giulio Natta”, CNR, Via Mario Bianco n. 9, 20131 Milan, Italy

**Keywords:** Enzymes, Fish feed, Aquaculture, Feed additive, Enzyme microencapsulation

## Abstract

Modern aquaculture must be sustainable in terms of energy consumption, raw materials used, and environmental impact, so alternatives are needed to replace fish feed with other raw materials. Enzyme use in the agri-food industry is based on their efficiency, safety, and protection of the environment, which aligns with the requirements of a resource-saving production system. Enzyme supplementation in fish feed can improve digestibility and absorption of both plant- and animal-derived ingredients, increasing the growth parameters of aquacultural animals. Herein we summarized the recent literature that reported the use of digestive enzymes (amylases, lipases, proteases, cellulases, and hemicellulases) and non-digestive enzymes (phytases, glucose oxidase, and lysozyme) in fish feed. In addition, we analyzed how critical steps of the pelleting process, including microencapsulation and immobilization, can interfere with enzyme activity in the final fish feed product.

**Supplementary Information:**

The online version contains supplementary material available at 10.1007/s42995-022-00128-z.

## Introduction

Intensive aquaculture requires effective and economical fish feed for growth of aquatic species in all life stages. The development of fish feed composed of all essential nutrients balanced to allow profitable growth, survival, and reproduction is needed (Sampath et al. 2020). In many fish feed formulations, protein and lipids are obtained from animal or plant sources (often by-products of the food industry) rather than through fish meal (FM) and fish oil (FO), which are being used in decreasingly smaller percentages in fish feed formulations. Even though some commercial fish feed uses alternative raw materials of non-marine origin that have good nutritional properties, they do not meet the unique nutritional value of feed formulated with only FM and FO. Hence, it is important to improve the availability and nutritional value of alternative raw materials through bioengineering technology specifically enzyme technology.

The use of enzymes is important for the development of a sustainable aquaculture industry (Son and Ravindran 2012). The global animal feed enzymes size reaches 1340.6 million USD in 2021, and it is expected a compound annual growth rate (CAGR) of 5.0% in the period 2022–2028, according to Global Animal Feed Enzymes Market Report (LP Information, Inc., 2022). These statistics suggest enzymes are becoming an important ingredient in the fish feed industry.

Fish feed must be optimally digested by appropriate enzymes to provide the required amounts of calories and essential nutrients to farmed fish. Most ingredients added to fish feed, especially non-fish raw materials, are composed of high molecular weight organic matter, which leads to slow decomposition and digestion in the digestive tract in fish. Several studies have shown that the use of enzymes for the pretreatment of plant-derived raw materials improved the fish feed digestibility and fish growth rate (Ai et al. 2007; Cao et al. 2007; Kalhoro et al. 2018; Maas et al. 2018, 2019, 2020; Ogunkoya et al. 2006). Most enzymes used in fish feed belong to a class of hydrolases, and, among these, proteases, glucosidases, and lipase have the highest number of applications (Ghosh et al. 2019). These enzymes can improve the digestion of antinutritional (ANT) factors present in fish feed, such as antigen proteins, indigestible oligosaccharides like stachyose and raffinose, and phytic acid, which cause slow digestion, malnutrition, and limited growth of fish. Dietary supplementation of phytases, essential for the digestion of plant-derived phytates, increases the bioavailability of phosphorus and other minerals, and overall growth performance (Lemos and Tacon 2017). In addition, to reduce aquaculture diseases and promote the health of farmed fish, some enzyme preparations used to improve intestinal health and inhibit harmful bacteria have also received attention, which is of significance for the control and reduction of antibiotic use, improvement of the environment, and food safety in aquaculture. Wherein, glucose oxidase and lysozyme are the most commonly used exogenous enzyme feed additives.

**Fig. 1:**
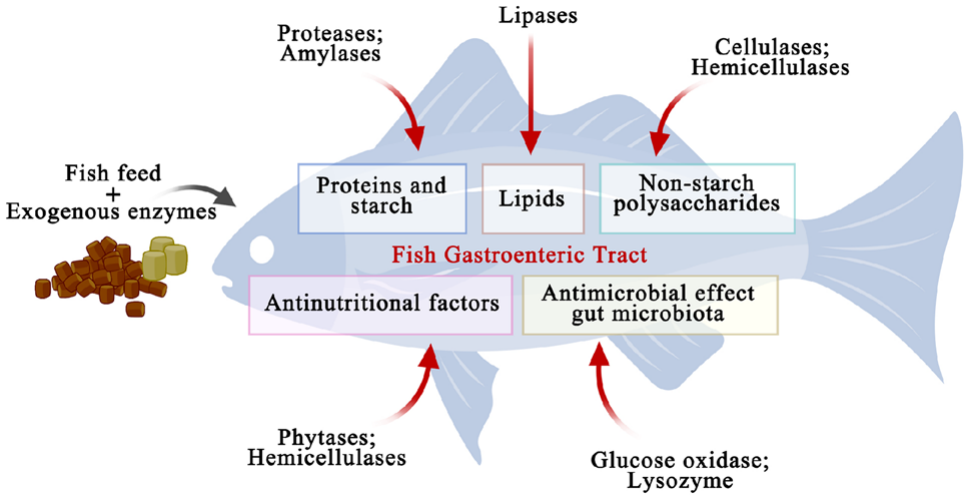
Representation of the possible roles in the gastroenteric fish tract of exogenous enzymes administered by fish feed and of the homologous endogenous enzymes (if present) after the ingestion of the feed

**Fig. 2:**
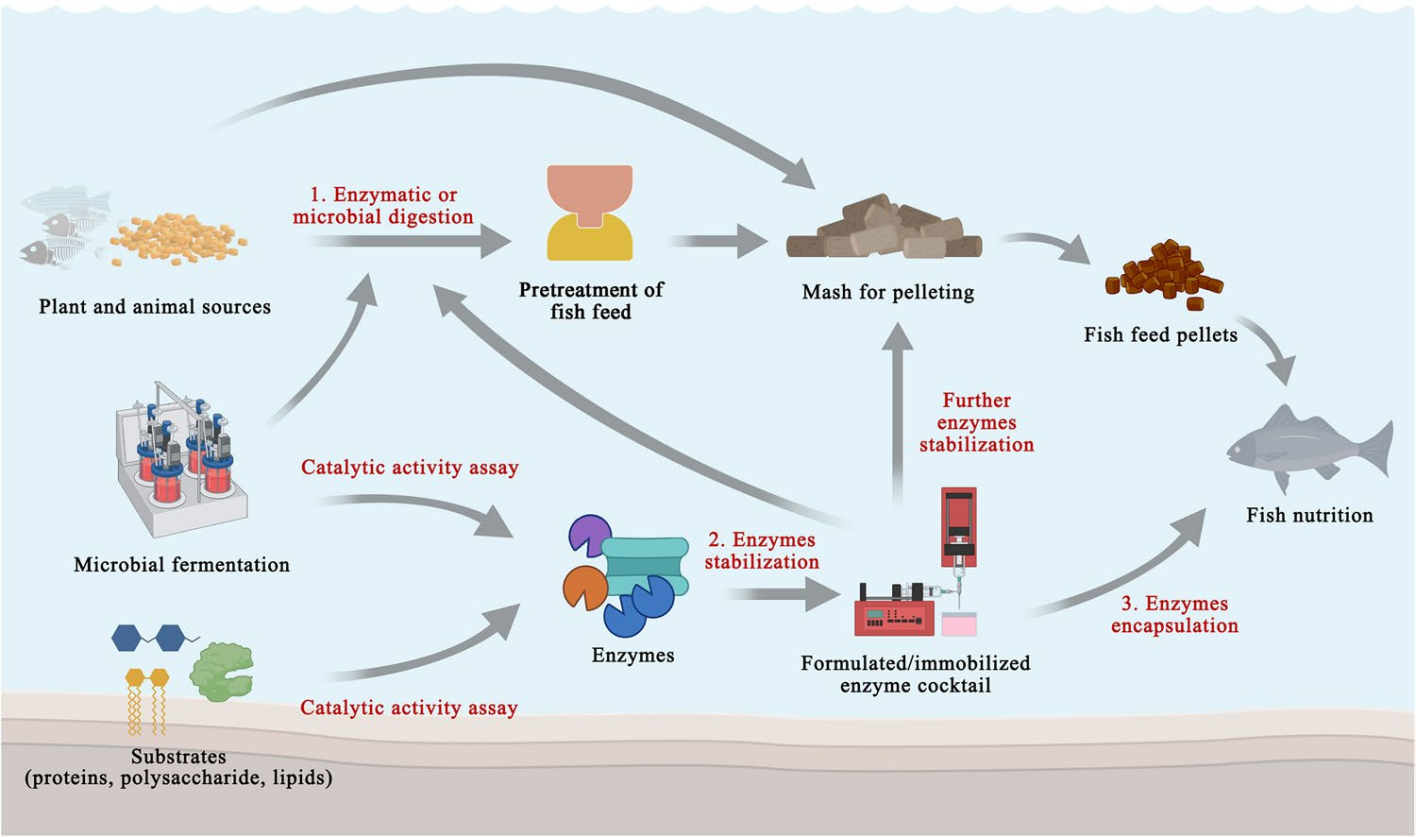
Origin, preparation, and possible points of the application of enzymes, in the pathways of fish feed production. Numbers 1, 2 and 3 indicate possible points where enzymes can act to (1) pre-digest fish feed, (2) be mixed with the mash for pelleting after enzyme stabilization toward temperature and operational conditions for pelleting or (3) be mixed with fish feed pellets to function in the gastroenteric fish tract

In this review, the three types of exogenous enzyme preparations mentioned above have been summarized and their application progress elaborated. In Supplementary Table S1, the main exogenous enzymes exploitable for aquaculture feedstuff and their application effects are listed. By presenting examples of the enzymes used in fish feed (Fig. 1) and their effect on fish growth performance, we highlighted critical issues to be considered for the efficient addition of enzymes to fish feed. Stability and high catalytic activity of fish feed ingredients remains a critical issue when using exogenous enzymes efficiently in fish feed formulations. These are listed in Fig. 2.

## Enzymes added in fish feed

### Proteases

Proteases are specific to the hydrolysis of peptide bonds located in the middle (endopeptidases) or at C- or N-terminus of the protein (exopeptidases). Also, some proteases are very selective by attacking only a particular amino acid sequence. Most protease applications in human food technology (Tavano et al. 2018) can also be useful to the fish feed industry. In particular, they can hydrolyze food proteins to peptides, enhancing their digestibility. Thus, exogenous proteases can supplement a low level of secretion in the fish digestive tract, assisting the endogenous enzymes to completely digest nutrients, improving their utilization.

Recent findings indicated that exogenous proteases have a significant effect on fish health including feed conversion ratio (FCR), weight gain (WG), and metabolic activity. The addition of exogenous protease into soybean meal has significantly increased the apparent digestibility coefficients (ADC) of rainbow trout (*Oncorhynchus mykiss*), varying from 0.792 to 0.869 (*P* < 0.05) (Dalsgaard et al. 2012). Adding exogenous proteases into a fish meal-free diet to be fed to tilapia (*Oreochromis niloticus* × *Oreochromis aureus*) increased WG from 1052.8% ± 24.4% to 1169.8% ± 11.4% (Li et al. 2019). A similar trend was reported when exogenous protease was added to an FM diet for Gibel carp (*Carassius auratus gibelio*), allowing an increase of WG from 188.4% ± 14.5 to 230.6% ± 6% (a pelleted diet) or from 244.5% ± 11.3% to 273.4% ± 6.0% (an extruded diet) (Shi et al. 2016). Supplementing protease in a diet with reduced FM level and crude protein content by 10% and 5% did not negatively affect the growth of white shrimp (Li et al. 2016). In this case, WG was 247.9% ± 11.3% for the control dietary group and 275.1% ± 6.2% for the diet containing 0.175 g protease per kg of feed. Furthermore, using protease was also very useful to improve the metabolic activity of the fish. Hassaan et al. (2019) compared the growth of juvenile Nile tilapia fed for 12 weeks with feed containing different ratios of FM/cotton seed meal (CSM) with or without the inclusion of exogenous protease (250 U/kg feed). In all cases, the supplementation of the protease in the diet led to the highest WG, protein efficiency, and feed efficiency. Enzyme addition also led to higher expression of insulin-like growth factor I gene in Nile tilapia brain and liver as compared to other fish groups fed with the same FM/CSM ratio but with no protease.

In the above-mentioned addition of exogenous protease to a fish diet, the effect varied according to the form in which the enzyme was added. A study by Dalsgaard et al. (2012) revealed that adding the same protease and β-glucanase to different feed had different effects, based on nitrogen mass-balance and energy retention data, with utilization of nutrients and energy better for the feed containing soybean. Furthermore, the use of proteases did not have a significant effect on growth parameters and FCR in rainbow trout when mixed and pelleted with a milled, sieved, dehulled, toasted, and solvent-extracted soybean meal fine powder (Yigit et al. 2018). This may be due to the different composition of the basal diet and processing modes having an impact on the action of exogenous proteases.

The most widely used proteases are neutral and alkaline, such as alkaline serine endopeptidase AG175TM from JEFO Nutrition Inc. of Saint-Hyacinthe, QC, Canada (Li et al. 2019; Shi et al. 2016). To date, acid proteases have also been shown to facilitate fish growth (Zheng et al. 2020). In addition, bromelain (an extract from the stems of pineapples containing proteases) could serve as a potent enzyme-based supplement to improve protein digestibility of spirulina-based fish diets, an economical alternative protein source of fish feed (Sharma et al. 2021).

The combination of exogenous proteases and endogenous digestive enzymes can be beneficial for fish growth. Fish produce endogenous enzymes that originate from their gastrointestinal system or gut microbiota. The latter one produces numerous digestive enzymes (Ray et al. 2012), such as cellulases, collagenases, proteases, amylases, allowing the digestion of complex organic macromolecules usually present in plant feedstuffs (Kar et al. 2008; Sinha et al. 2011). The fermentation of engineered microbial strains is the main route for producing exogenous enzymes at an industrial scale. Also, by-products produced from the fish industry (e.g., fish viscera) could be a potential source of enzymes to be used as additives in aquafeed (González-Riopedre et al. 2013).

### Amylases

Starch is the main digestible polysaccharide in plant feed ingredients used in aquaculture (Francis et al. 2001). The digestibility of starch directly affects the growth of fish. Therefore, amylase, an enzyme that degrades starch (Upreti et al. 2019), plays a crucial role in the growth process of fish. Although amylase is an endogenous digestive enzyme of numerous fish species, some studies have shown that carnivorous fish could have a low expression of this enzyme, resulting in a reduced ability to digest starch for energy supply (Hemre et al. 2002; Stone 2003). Moreover, carnivorous fish might suffer prolonged postprandial hyperglycemia (Bergot 1979; Moon 2001; Wilson 1994), low metabolic activity, reduced utilization rate of other nutrients in fish feed with a high content of dietary carbohydrates (e.g., starch, dextrin) (Hemre et al. 2002), and fish stress response (Petitjean et al. 2019).

In response to the afore-mentioned problems, studies have shown that adding exogenous amylase could increase metabolic activity and regulate blood glucose levels. Kumar et al. (2006a, b) studied the addition of α-amylase to the feed of *Labeo rohita* (Hamilton). The authors reported that the addition of 50 mg α-amylase/kg feed led to an increase of liver glycogen (from 97.13 ± 1.29 mg to 171.36 ± 3.19 mg glycogen g^–1^ wet tissue) (Kumar et al. 2006b) and blood glucose (about 80 to 102 mg glucose in 100 ml blood), meaning that under the effect of exogenous amylase, the starch utilization and glucose metabolism in the fish was increased. In follow-up studies, Kumar et al. (2009) reported that glucose-6-phosphate 1-dehydrogenase (G6PD) activity of the fish was also enhanced, suggesting amylase supplementation could enhance metabolism and regulate postprandial blood glucose. Interestingly, these authors also revealed a complicated metabolic situation. After adding the same amylase to feed with different protein levels (gelatinized or non-gelatinized corn containing optimum (35%) or sub-optimum (27%) protein levels), the non-gelatinized corn diet appreciably enhanced gluconeogenetic and amino acid metabolic enzyme activity, whereas gelatinized corn induced increased lipogenic enzyme activity in the serum and liver of fish that can be correlated to the type of corn and protein level.

Some studies reported that adding exogenous amylase to fish feed also improved the protein content, health status, and immunity of fish. Elevated fish muscle protein, muscle protein/DNA ratio, and DNA/muscle mass (wet weight) ratio were all used in studies exploring the effects of exogenous amylases on fish (Khalil et al. 2018; Kumar et al. 2006a, b, 2009). Khalil et al. (2018) also reported that when adding a single amylase to the feed of striped catfish (*Pangasianodon hypophthalmus*), the red and white blood cells count, hematocrit, and numbers of lymphocytes along with other haematological parameters representing the immune activity of the fish, improved. In addition, a decreasing tendency of the total bacterial count in the intestinal content and fish skin was observed. These results proved that exogenous amylase could play a role in enhancing fish immunity and improving fish health.

At present, only a few studies examined the effects of exogenous amylase on fish, and most focused on diets supplemented with mixed enzyme preparations containing amylase. Those studies showed that growth performance could be improved by the addition of exogenous enzymes. For example, the addition of pepsin, papain, and α-amylase into the feed for Nile tilapia (*O. niloticus*) fingerlings led to a significant increase of WG, and feed utilization (Goda et al. 2012). Similarly, Atlantic salmon, *Salmo salar* L. (a carnivorous fish) showed an enhanced feed utilization rate after receiving exogenous α-amylase through feed, and flesh quality was more appreciated by fish consumers (Carter et al. 1992, 1994). Yildirim and Turan (2010) added a commercial preparation containing β-amylase to the feed of African catfish (*Clarias gariepinus*). The use of this enzyme, along with protease, fungal xylanase, β-glucanase, endo-β-glucanase, pentosonase, α-amylase, and pectinase, improved fish growth and the specific growth rate (SGR). However, the authors did not provide details on the enzyme specific activity, making it difficult to establish which of these biocatalysts was responsible for the positive effect on fish growth.

### Lipases

Lipases are carboxylic ester hydrolytic enzymes, which sequentially hydrolyze the ester bonds of triglycerides to form glycerol and fatty acids. Lipases and phospholipase A2 are the two types of lipolytic enzymes that have been mostly studied in fish metabolism (Iijima et al. 1998; Zambonino and Cahu 2007). They have a crucial role in modulating fish adipose tissues, ultimately affecting carcass yield and flesh quality of farmed fish species (Weil et al. 2013). The oral cavity of fish larvae often contains lipases (Murray et al. 2003; Srivastava et al. 2002), but bile salt-activation is usually required to activate their function (Iijima et al. 1998; Murray et al. 2003). Because juvenile fish have a better ability to digest phospholipids than triglycerides, and the pancreatic lipase of juveniles is non-linear in the digestive level of dietary triglycerides (Cahu et al. 2003), lipases have been added as a feed component to increase the level of lipid digestion. Studies on lipases as an exogenous enzyme added to fish feed and their effects on fish performance are still relatively few and primarily conducted using mixtures with other enzymes.

Some studies have demonstrated that the multienzyme commercial preparation containing lipase improved fish growth performance. Ghomi et al. (2012) added lipase to the feed of great sturgeon *Huso huso* fingerlings. As a result, their SGR (from 3.32 ± 0.19 to 3.68 ± 0.17) and final weight (from 46.13 ± 0.20 g to 53.03 ± 0.15 g) increased. Similarly, Zamini et al. (2014) added a multienzyme commercial mixture containing lipases to the diet of Caspian salmon (*Salmo trutta caspius*). The survival rate and average body weight of fish were all higher than the control group, with an increased rate of 5.85% and 24.06%, respectively. These studies confirmed that exogenous lipase can contribute to increased fish growth by increasing the SGR.

Adding exogenous lipase to fish feed could also improve the quality of fish meat. After feeding fish with feed containing exogenous lipase, the fat content in the carcass of great sturgeon *H. huso* fingerlings significantly increased (34.53% ± 0.06%, control 27.83% ± 1.75%) (Ghomi et al. 2012). The same study also showed that the content of n-3 essential fatty acids of fingerlings fed with 500 mg/kg enzyme was higher (5.05% EPA and 5.89% DHA) than the control fish, which had 1.52% and 4.12% EPA and DHA content, respectively.

Some studies have also shown that adding lipase to feed can enhance fish metabolic activity. Liu et al. (2016) supplemented lipase into the diet of young grass carp (*Ctenopharyngodon Idella*) (average initial weight 255.02 ± 0.34 g) and reported an increase in intestinal weight (16.67 ± 1.55 g/fish instead of 11.83 ± 1.33 g/fish measured in the controls), feed efficiency (varied from 57.67 ± 1.22 for the control to 63.74 ± 1.48), and intestinal immunity, indicating an improvement in the anti-inflammatory response. Furthermore, the addition of lipase led to an increase of interleukin 10 and acid phosphatase mRNA copies in the intestinal tract (about 7–8%). Other positive effects were the up-regulation of the mRNA copies of genes encoding for the antimicrobial peptides, anti-inflammatory cytokines, and antioxidant enzymes (e.g., copper/zinc and manganese superoxide dismutase, catalase, peroxidase, S-transferases, and glutathione reductase).

In addition to adding exogenous lipase to fish feed, the addition of a large amount of lipase produced by microbial fermentation is also an effective way to promote lipid decomposition in fish feed. *Yarrowia lipolytica* is the most widely used microorganism at present, proven to play a role in promoting the growth of fish when added to fish feed. *Y. lipolytica* added to the diet significantly increased the growth rate and body weight of Russian sturgeon (*Acipenser gueldenstaedtii*), and Atlantic salmon, as well as the concentration of EPA and DHA in their muscle, indicating the nutrient content and taste of farmed fish could be improved (Chen et al. 2021; Hatlen et al. 2012). The present results suggest that *Y. lipolytica* could be used as a potential diet additive for aquaculture.

According to these studies, lipases might have a series of beneficial effects when administered to fish via a low-protein and high-lipid diet. More studies on the use of these enzymes, in combination with microbial products are needed.

### Cellulases

Cellulases hydrolyze β-1,4 glycosidic bonds in the polymer to release glucose units allowing the use of cellulase as a source of carbohydrates that provides energy to the body (Barr et al. 1996). Cellulase activity has also been detected in the gastrointestinal tract of some fish (Stickney and Shumway 1974), and it has been demonstrated that the cellulase activity is mainly contributed by the gastrointestinal microbial community of the fish rather than by the fish itself (Lindsay and Harris 1980; Saha and Ray 1998).

The level of cellulose in fish feed affects the utilization of other nutrients. The lack of cellulase and the absence of a stomach in some fish species can be responsible for the low digestibility of cellulose. Cellulose and other non-starch polysaccharides (e.g., pectins, galactans) might have adverse effects on nutrient absorption because of the binding to bile acids, the obstruction of digestive enzymes, and the movement of substrates in the intestine (Francis et al. 2001).

European sea bass (*Dicentrarchus labrax*), rainbow trout (*O. mykiss*), rainbow trout (*Salmo gairdneri*), and Nile tilapia (*O. niloticus* L.) fed with high levels of cellulose in the feed have shown low intestinal absorption rates, low utilization of nutrients, and decreased growth performance (Amirkolaie et al. 2005; Bromley and Adkins 1984; Buhler and Halver 1961; Davies 2019; Dias et al. 1998; Hansen and Storebakken 2007; Hilton et al. 1983). Depending on the fish species, the highest tolerable level of cellulose in the diet can vary from 10 to 30% w/w (Bromley and Adkins 1984; Dias et al. 1998).

Zhou et al. (2013) have shown that the addition of exogenous cellulase to grass carp feed could promote growth rate after 60 days of feeding (the WG ratio was 164.61% ± 0.51% and 177.30% ± 0.43% for the control and the group fed with a diet supplemented with cellulose, respectively). The fish feed used in this study was a mixture of shredded *Lemna minor* Linn. mixed with wheat flour (*L. minor* Linn. / wheat flour 10/1, w/w). The supplemented cellulase was from *Trichoderma longibrachiatum* (SIGMAC9748, USA) with specific activity greater or equal to 1.0 U/mg. The cellulase was added at a ratio of 3 g/kg of duckweed (corresponding to 3000 U/kg) and mixed with wheat flour. Additionally, the activity of the grass carp digestive enzymes (e.g., protease and amylase) for the group fed with a diet enriched in cellulase increased in comparison to the control group. In particular, the activity values were 2.10 ± 0.10 (control group 1.65 ± 0.02), 99.43 ± 2.42 (control group 58.45 ± 2.19) U mg prot^−1^ for amylase, 29.57 ± 1.15 (control group 24.00 ± 1.12) U μg prot^−1^ for protease, and 27.12 ± 0.57 (control group 25.54 ± 1.59) U g prot^−1^ for the lipase.

Some recent studies indicated that cellulases have a significant effect on the gut microbiota of fish. The study of Zhou et al. (2013) highlighted that the increase of *Bacillus* and *Sphingomonas* in the gut microbiota of grass carp contributed to the digestion of cellulose. The supplementation of cellulase increased the number and abundance of bacterial species of carp gut microbiota, which was beneficial for the digestion of nutrients and played a key role in the immune response and disease resistance of fish (Burr et al. 2005). In general, carnivorous fish might need more exogenous cellulases in the feed (Zhou et al. 2013), as they have fewer bacteria involved in cellulose digestion in comparison to herbivorous fish. However, some researchers have isolated a bacterial strain with cellulase activity in the gut microbiota of grass carp (Li et al. 2009), but there were no reports on similar strains isolated from the intestinal tract of carnivorous fish.

Although some studies have shown that the addition of exogenous cellulase to carnivorous or herbivorous fish could promote fish growth (about 15% increase of the final fish body weight and 5% of SGR) (Ai et al. 2007; Ghomi et al. 2012; Zhou et al. 2013), the effect of dietary cellulase on fish growth was not always positive (Carter et al. 1992; Ogunkoya et al. 2006). The absence of beneficial effects may be due to a variety of factors, such as fish species, type of fish feed, way of enzyme addition, and aquaculture environment. For instance, the addition of 20,000 U/kg cellulase to the diet containing canola meal (CM) fed to tilapia did not promote fish growth and nutrient digestibility at any of the CM concentrations tested (Yigit and Olmez 2011). Moreover, the addition of different proportions of cellulase to rapeseed diets did not affect the growth parameters and nutrient digestibility of *Pterophyllum scalare* (Erdogan and Olmez 2009). Most cellulases in fish are not produced endogenously but derived from bacteria and fungi present in the feedstuffs. The pH of the fish digestive environment (different from the optimal pH for enzyme activity) may affect the efficacy of dietary cellulases (Zhou et al. 2013). Furthermore, the high content of cellulose in the feed may also affect the digestive activity of proteases. For example, the high levels of fibre, either alone or together with phytate, had the greatest adverse effects on the digestibility of canola protein products in rainbow trout (Mwachireya et al. 1999).

Cellulase catalytic activity is of great interest for the fish feed industry especially when plant-derived raw materials are used because many fish species lack the ability of cellulose degradation (Opuszynski and Shireman 1995). However, to date, the use of this type of enzyme in fish feed has not been sufficiently studied, so further research in this area is needed.

### Hemicellulases

Hemicellulases include a group of enzymes involved in the breakdown and hydrolysis of galactans, xylans, mannans, and arabans (Chadha et al. 2019). To reduce production costs, non-starch polysaccharides (NSP), such as wheat, grains, and bran, have been used as the main energy source for fish feed, affecting the absorption of nutrients by fish. Oligosaccharides, such as xylo-oligosaccharide, mannan oligosaccharide, fructo-oligosaccharide, and galacto-oligosaccharide, produced by hemicellulose degradation of the above-mentioned raw materials have certain probiotic activities, which not only improve the nutrient absorption of cultured animals but also improve the intestinal health of animals. Therefore, supplementation of exogenous hemicellulases in fish feed has become the main way to solve the problem of digestion of NSP and to facilitate the growth of fish. Currently, the hemicellulases used as an additive in the aquafeed industry mainly include xylanases and glucanases. Several studies have shown that the addition of β-xylanases and β-glucanases to fish feed can improve fish growth rate and feed utilization, enhancing the quality of fish. These observations were reported for Atlantic salmon (*S. salar*) (Jacobsen et al. 2018), silver perch (*Bidyanus bidyanus*) (Stone 2003), tilapia (*O. niloticus* × *O. aureus*) (Lin et al. 2007; Maas et al. 2018, 2020), African catfish (*C. gariepinus*) (Yildirim and Turan 2010), and shrimp (*Litopenaeus vannamei*) (Qiu and Davis 2017).

It has been reported that different methods of adding exogenous hemicellulases in fish feed can also affect fish growth rate. To promote the synthesis of xylanase, the addition of xylanase-expressing *Bacillus amyloliquefaciens* R8 to fish feed improved the growth performance of Nile tilapia (Saputra et al. 2016). After two months of the feeding trial, the final weight of Nile tilapia was 17.7 ± 0.19 g, starting from an initial fish weight of 1.5 ± 0.0 g, with the control group reaching a final weight of 9.0 ± 0.33 g after the same feeding time. Xylanase addition also increased the metabolic activity of the tilapia liver, determined as the relative mRNA expression level of growth- and metabolism-related genes of glucokinase (GK), glucose-6-phosphatase (G6Pase), G6PD, and insulin-like growth factor-1 (IGF-1). These enzymes increased about three- or fourfold, as compared to the control tilapia fed on a diet without the addition of *B. amyloliquefaciens R8*. The evidence of an enhanced metabolic activity supports the increased growth rate in fish fed with *B. amyloliquefaciens* R8 added to the diet. Furthermore, lysozyme activity of *B. amyloliquefaciens* R8-fed Nile tilapia was noticeably higher (1.1 mg/ml) than in fish fed with the control diet (0.39 mg/ml), suggesting an enhancement of the resistance of tilapia to *Aeromonas hydrophila* (Saputra et al. 2016).

Interestingly, the addition of mixed exogenous enzymes may affect the metabolites of fish without affecting the growth of the fish itself. Ogunkoya et al. (2006) added a commercial enzyme cocktail containing xylanase, amylase, cellulase, protease, and β-glucanase (Superzyme CS, Canada Bio-system Inc., Calgary, Alberta, Canada) at a ratio of 1 or 2.5 g/kg feed in the diet of rainbow trout (*O. mykiss*). Although the enzyme cocktail supplementation (no indication of specific activity was reported) did not affect the growth of *O. mykiss*, it reduced faecal material cohesiveness and sinking speed, which can potentially minimize waste recovery on land-based fish aquaculture operations and the impacts of some cage culture plants.

### Phytases

Phytate is an antinutritional factor widely found in plant-based feed raw materials. It forms chemical complexes with mineral elements (e.g., calcium, iron, magnesium, and zinc), proteins, and other nutrients, decreasing the absorption and utilization of these substances (Humer et al. 2015). In particular, phytate-bound phosphorus has very low bioavailability in monogastric terrestrial animals, such as pigs or fish, due to the absence of an intestinal phytase in these animals (Adeoye et al. 2016). Due to the increasing number of commercial phytases capable of effectively degrading the antinutritional factors, it is becoming common practice to add phytase to fish feed.

In recent years, phytase has been used more frequently in fish feed (Eyiwunmi et al. 2017; Lemos and Tacon 2017) showing an impact in different physiological processes. Some studies have shown that the addition of phytase has an impact on the bioavailability of phosphorus and environmental safety. Phosphorus is an essential mineral for fish growth, but its release in the environment is increasing, and it may be responsible for pollution and eutrophication of watersheds and coastal seawater (Bohn et al. 2008). The use of exogenous phytase is substantially efficient in reducing phosphorus excretion by converting phytate phosphorus into bioavailable free inorganic. Morales et al. (2016) and Olugbenga et al. (2017) found that using plant-based diets supplemented with phytase led to a reduction of 50% and 31% of phosphorus loadings in rainbow trout and catfish, respectively. Chen et al. (2019) have also reported the positive effects of phytase in increasing WG (from 772.27% ± 5.52% to 1027.25% ± 32.34%). These results indicated that phytase might play an essential role in decreasing the dispersion of phosphorous in the surface waters or in the coastal environments and, consequently, in reducing the risk of eutrophication.

It has been pointed out that exogenous phytase in feed could improve the growth performance of fish. Indeed, several studies showed that dietary phytase could significantly improve the growth performance and feed utilization in channel catfish (*Ictalurus punctatus*) (Chen et al. 2019), African catfish (*C. gariepinus*) (Kemigabo et al. 2018), Nile tilapia (*O. niloticus*) (Adeoye et al. 2016), and Pacific white shrimp (*L. vannamei*) (Pakravan et al. 2017). Different doses of phytase (150 to 2000 FTU (phytase units)/kg) added in the fish feed increased weight and growth rate to different levels (Debnath et al. 2005). It was found that the optimum dose of added phytase in the catfish was almost 300 FTU/kg (Rachmawati and Samidjan 2018), with a similar result found in giant tiger prawns (Rachmawati and Samidjan 2016). However, Shahzad et al. (2020) found that the mixture of Moringa seed meal and Moringa leaf meal-based diet supplemented with exogenous phytase at 900 FTU/kg concentration was suitable to develop a cost-effective and eco-friendly fish feed with maximum absorption of important nutrients and improvement of the overall performance of *Catla catla* fingerlings. The dose and concentration of phytase added to fish feed may be determined according to the type of fish feed and the species of fish to maximize the benefits of exogenous enzyme supplementation in fish aquaculture.

In addition, microbial phytase has been shown to play a critical role in the bioavailability of nutrients for different fish species. Using a plant-based diet, Morales et al. (2016) observed that apparent digestibility and utilization of P, Ca, Mg, and Zn in rainbow trout (*O. mykiss*) were improved by the use of phytase. The results reported by Akpoilih et al. (2016) showed that using phytase in plant-based diets resulted in a 13% and 50% reduction in N and P loadings, respectively. Moreover, in the presence of phytase, the percentage of N and P intake was 51.2 ± 0.8 and 68.5 ± 3.6, respectively, whereas, for the control, it was 48.8 ± 0.3 and 37.2 ± 2.2, respectively. The addition of phytase and sodium diformate (NaDF) in the diet led to a higher final fish weight than in the control fish fed a diet without these exogenous enzymes. Moreover, those studies reported that ADC of protein and amino acids significantly increased when 500 and 1,000 IU/kg of phytase was included in the feed of shrimp or giant tiger prawn (Bohn et al. 2008; Rachmawati and Samidjan 2016). Nevertheless, although numerous studies have demonstrated the great benefit of phytase as an additive for aquaculture feeds, further research needs to confirm the optimal dose, species specificity, and the role of phytase.

### Glucose oxidases

Glucose oxidase (GOD; E.C.1.1.3.4.) is a flavoprotein that catalyzes the dehydrogenation of β-D-glucose to gluconic acid and hydrogen peroxide (H_2_O_2_) by utilizing oxygen (O_2_) as an electron acceptor (Hatzinikolaou et al. 1996). GOD is widely diffused, particularly in microorganisms, including *Penicillium* and *Aspergillus niger* species (Bhat et al. 2013; Eryomin et al. 2004; Todde et al. 2014), such as *Penicillium pinophilum, P. amagasakienses,* and *P. funiculosum*. Most GOD that is commercially available is produced by microbial strains that have an optimum pH range of 5.0 to 7.0, a factor that must be considered when using this enzyme in fish feed formulations (Bankar et al. 2009). Microbial GOD is currently receiving significant attention due to its widespread use in the chemical, food, beverage, feed, biotechnology, and other industries.

GOD used as a feed additive has been shown to promote fish growth and enhance health. According to recent reports, a diet supplemented with GOD increased the levels of growth and development-related hormones in piglets (Biagi et al. 2006; Tang et al. 2016; Wang et al. 2018), improving the feed conversion rate and growth performance. GOD preparation can also quickly remove bacterial toxins and intracellular toxin poisoning in animals. GOD is a safe and pollution-free alternative to antibiotics. Jeong et al. (1992) reported that GOD added to a poultry compound feed enhanced egg production and inhibited various moulds, such as *Aspergillus flavus*, *Rhizopus oryzae*, and *Penicillium*.

Some studies have also showed that exogenous GOD can improve the intestinal acidic digestive environment and contribute to maintaining a balanced intestinal microbiota. This role can be attributed to the gluconic acid produced by glucose oxidase, resulting in a partial acid environment to enhance intestinal health (Biagi et al. 2006). Furthermore, by consuming oxygen in the intestine GOD creates an anaerobic environment for the proliferation of beneficial anaerobic bacteria (Bankar et al. 2009), whereas the hydrogen peroxide produced can inhibit the growth of *Escherichia coli* and *Salmonella*.

In addition, the most unique aspect of adding glucose oxidase to feed is that it can guarantee the quality of raw materials and feed. For instance, GOD addition to the feed consumed oxygen (Hatzinikolaou et al. 1996), inhibiting the growth of aerobic microorganisms and preventing spoilage.

Though the beneficial effects of GOD supplementation are evident in pigs (Tang et al. 2016), its use in fish feed is still in the early stages. From the perspective of avoiding the use of antibiotics in aquaculture, GOD may have a broad application in the feed industry.

### Lysozymes

Lysozyme can act on the β-1,4-glycosidic bond of bacterial cell walls and has certain bactericidal effects. It is ubiquitously present in animal body fluids as a non-specific antibacterial factor. Lysozyme was originally extracted from egg white by a complicated and expensive procedure. At present, lysozyme can be produced on an industrial scale by *Pichia pastoris* and other engineered strains, greatly reducing the cost of its application in feed.

The haematological indices including white blood cell, red blood cell, and hematocrit of rainbow trout (*O. mykiss*) fingerlings were significantly improved with the effect of dietary lysozyme in fish feed. Instead, there was no significant increase in the growth performance in fish fed with different levels of dietary lysozyme (Shakoori et al. 2018). Furthermore, utilizing lysozyme conjugates with galactomannan or palmitic acid as a therapeutic for infection in fish, the survival rate was increased after supplementing the dietary lysozyme to the *Edwardsiella tarda*-infected carp, *Cyprinus carpio* L. (Nakamura et al. 1996). Lysozyme-galactomannan conjugate was prepared through a controlled Maillard reaction and lysozyme-palmitic acid conjugate was prepared through base-catalyzed ester exchange using N-hydroxysuccinimide ester of palmitic acid. The above results showed the possibility of utilizing lysozyme conjugates with galactomannan or palmitic acid as an infection therapeutic in fish. Therefore, the addition of lysozyme in fish feed and the effective production methods facilitate the development and application of this type of enzyme as a feed additive.

## Enzyme addition procedures

One main issue when selecting an enzyme to be included in fish feed is its ability to transform complex feed components into absorbable nutrients. For this reason, enzymes can be added even if the feed preparation includes a fermentation process. For example, lactic acid bacteria are the most commonly applied bacterial agent in the process of soybean meal fermentation, due to their ability to produce delightful flavour (Tsai et al. 2021). However, lactic acid bacteria are less capable of producing proteases that allow the hydrolysis of proteins into smaller peptides during fermentation. Furthermore, the removal of the antigenic proteins present in soybean meal fermented by lactic bacteria is very difficult. To overcome this difficulty, exogenous proteases are usually added during soybean meal fermentation (Jiang et al. 2021).

Nutritional enzymes are generally added to feed, while antibacterial enzymes (e.g., glucose oxidase) may be added to animal protection products, not necessarily mixed into feed. The pretreatment of the feed components can improve nutrient utilization, reducing the excretion of nutrients into the environment. However, feed enzymatic pretreatment is rarely used as it increases feed cost and can have adverse effects on the feed properties (e.g., microbial contamination) and on the final pellet characteristics (e.g., loss of firmness and texture). Therefore, for the various type of exogenous enzymes considered in this review, the methods of addition should be selected according to their characteristics in the preparation of aquatic feed. Some studies indicated that the use of enzymes (e.g., carbohydrase or protease) did not show any improvement in nutrient digestibility or fish growth (Carter et al. 1992, 1994; Denstadli et al. 2011; Ogunkoya et al. 2006; Yigit and Keser 2016), but an opposite outcome was observed with the same enzymes employed in salmonid diets.

The incongruent effect of these enzymes used in the diet of different types of fish could originate from the use of different ingredients, types of enzymes, other procedures used for their addition in the feed, and rearing conditions, such as water temperature and stage of growth of the fish. Thus, the multiplicity of the parameters that might influence the results of using enzymes in fish feed requires a precise and accurate set-up of the experiments not only in terms of fish nutrition but also in the preparation of the fish feed itself.

Enzymes have optimal functionality with appropriate operational conditions. In the case of their use to digest fish feed ingredients (e.g., proteins and polysaccharides) or to improve absorption of nutrients, their catalytic activity can change according to the conditions of fish gastroenteric apparatus. It is worth noting that farmed fish species may not have a functional stomach that can digest nutrients during larval stages. The digestive apparatus and its functionality gradually develop as fish larva age (Govoni 1980), but some species do not develop a functional stomach at all. Fish stomach pH is one of the most significant changes that occur during the growth of the animal. At first, the pH of the fish stomach is neutral or slightly alkaline (6.7–7.1), but as fish grow, the pH value gradually decreases and 97 days post-hatching, it can be as low as 5.0 (Mahr et al. 1983). For this reason, the acid resistance of enzymes added to fish feed should be considered differently depending on the developmental stage of fish. Thus, it appears that exogenous enzymes for most adult fish feed, need to have some acid resistance.

Beyond pH, other factors, such as temperature applied during fish feed processing and moisture level, can have a significant impact on enzyme activity. It is crucial to use processing techniques that maintain enzyme activity during the pelleting process. The procedure usually adopted for pelleting includes extrusion of conditioned hot mash through a suitable die of a defined length and diameter. Before being extruded, feed ingredients pass through the conditioner where they are treated with steam under pressure and exposing the mash to high temperatures before entering the pellet die (Amerah et al. 2011). The conditioner temperatures in feed mills may reach 95 °C, or even higher, to prevent growth of foodborne pathogens (Doyle and Erickson 2006; Jones and Richardson 2004). However, for this purpose, prolonged exposure to steam and increased steam pressure is required.

During feed processing, factors such as pressure, heat, time, and moisture level in the conditioner room might explain enzyme unfolding and inactivation (Silversides and Bedford 1999; Spring et al. 1996). Enzyme addition requires the development of specific procedures that protect enzymes in fish feed, especially if they have to be active in the gastroenteric tract of the fish. Thus, the pelleting process needs to ensure the retention of the enzymatic activities (Fig. 2, points 1–3).

Different procedures maintain a high level of enzyme activity in fish feed. Spring et al. (1996) reported that the catalytic activity of several enzymes can be maintained in pellets even if prepared at temperatures up to 80 °C or for bacterial amylase, up to 90 °C. Cellulase, pentosanase, and fungal amylase after pelleting showed a residual activity of about 80% at temperatures up to 80 °C, and at 90 °C the residual activity was 10%, 5% and 5% (compared to the control), respectively. In the same study, bacterial amylase maintained about 50% of its activity even when carrying out the pelleting at 100 °C. Moreover, the decrease of feed viscosity even after cellulase pelleting at 100 °C suggested that the enzyme was stable in the operational conditions of the process. The endolytic activity reduced viscosity, whereas the release of sugars from a soluble substrate was the result of exolytic activity. Hence, the authors concluded that the observed loss in enzymatic activity caused by pelleting might be a consequence of the analytical method used to assay the activity (unable to monitor the endolytic activity) and not because of the enzyme inactivation.

An alternative way of administering exogenous enzymes in a fish diet could be preparing the enzyme in a separate formulation. This strategy might protect enzyme function during the industrial process of aquafeed production and also from the activity of endogenous proteases and other physicochemical factors (e.g., pH) in the fish digestive tract. Many lipids and natural polymers employed as protein or drug carriers can potentiate the efficiency of exogenous enzymes in both medical and feed industries. Also, there is an ample choice of micro-encapsulation methods to select the most appropriate one for a specific enzyme and a given application (Ye and Chi 2018). Alginate, chitosan, and xylans have been successfully employed as gel matrices for effective enzyme immobilization by entrapment into particles (microencapsulation). The immobilized enzymes prepared in this way, often are more stable toward external inactivating factors (Sirisha et al. 2016).

Some examples of enzyme microencapsulation have been recently reported by Rodriguez et al. (2018). They prepared alginate and alginate-bentonite microcapsules for the intestinal delivery of shrimp proteases in Nile tilapia. The addition of bentonite to the gelling solution improved the capsule performance under different storage methods leading to better retention of the enzyme activity. Furthermore, the reference diet and alginate-bentonite capsules containing shrimp enzymes showed a 27% higher enzyme activity in the fish intestines than the reference diet. The authors concluded that this type of microencapsulation could represent a suitable carrier for delivering exogenous shrimp enzymes in farmed fish.

Yao et al. (2019) reported that micro-encapsulated protease and carbohydrase added into the feed for Pacific white shrimp did not improve hepatopancreatic lipase activity. Guo et al. (2020) observed that microencapsulation of exogenous enzymes led to a decrease in the amylase activity in shrimp hepatopancreas, which was in contrast with the upswing trend in trypsin at the pancreatic segment. Thus, microencapsulation may have adverse effects. A possible explanation is that the encapsulated enzymes were not able to act in the proximal intestine, where most of the digestion and absorption of nutrients occurs.

In a different study, the encapsulation of microbial phytase in chitosan/alginate-based microcapsules improved the apparent digestibility and bioavailability of nutrients from plant protein-based diets in rainbow trout, including a better growth performance and tissue mineralization. The study was performed with 300 FTU phytase/g microcapsules. However, encapsulation tended to diminish phytase ability to release phosphorous. After 42–56 days of feeding, feed efficiency was 0.75, 1.25, 0.95 and thermal growth coefficients were 0.110, 0.162 and 0.148 for a diet with i) no supplemental phytase, ii) with 3000 FTU non-encapsulated phytase/kg (as-fed basis), and iii) 3000 FTU encapsulated phytase/kg (as-fed basis), respectively. For the same type of diet, the P retained was, in the order, 2.92, 3.03, 3.12, and P intake was 6.7, 4.6, and 5.80, respectively. The authors ascribed this effect to a reduced interaction between the enzyme and dietary phytate-P (Vandenberg et al. 2011).

The points already discussed indicate that not only the enzyme but also its preparation and the method of enzyme administration to the aquatic farmed animals is of fundamental importance for optimal bioprocessing of the aquafeed. Although different procedures have proved to be useful for enzyme incorporation in the fish feed, they can vary according to the enzyme type, enzyme biochemical properties, and target aquatic species and their stage of growth. However, the vast number of enzymes and biocompatible materials commercially available, jointly with the numerous techniques developed for the handling and inclusions of enzymes in fish feed as well as in human food and pharmaceutical products, can certainly lead to the development of fish feed enriched with enzymes. At present, a conspicuous number of enzyme products are available in the market as additives for many types of applications, from food and detergent industry and aquaculture. In addition, the production of enzymes used in feed is usually conducted through large-scale manufacturing methods, such as engineered bacterial fermentation, which generally costs less, making them affordable for large-scale animal feed production.

For aquaculture purposes, there is a need for specific enzymes more active toward the components of the fish feed (non-starch polysaccharides, vegetable protein) and stable to the conditions of fish feed manufacturing (e.g., pelleting temperature). To this end, Bedford (2000) explored the use of genetic engineering to improve exogenous enzyme thermal stability and adaptation to the high temperatures of the process of feed preparation. Gordeeva et al. (2019) increased the thermal stability of enzymes used in feedstuffs by site-directed saturation mutagenesis. Genetic engineering has been widely used to improve enzyme properties to promote their better application.

## Conclusions

Modern aquaculture, underpinned in the principle of sustainability, requires alternative sources to FM and FO, which need to be transformed to reproduce as closely as possible to the natural fish diet. In this view, exogenous enzymes can be an essential component of fish feed to compensate for the lack of endogenous biocatalysts for the alternative raw materials in the feed. Several factors, such as the choice of the enzyme activity and enzyme properties, type of preparation, or encapsulation for administering it, conditions employed to include the biocatalyst in the aquafeed might cause the absence of positive effects on fish growth. Adding enzymes to fish feed has to take into account the numerous variables that the fish feed itself might contain. Noteworthy is the improvement of the feed by enzymes appears more efficient when a cocktail of enzymes is used instead of a single enzymatic type. Isolation of new enzymes, including those promoting digestion and growth, antimicrobial activity, repairing intestinal inflammation, or even improving the body's immunity, is the direction that needs to be considered in the future especially under the current trend of banning the use of antibiotics.

Secondly, enzymes still have problems that cannot be ignored, such as sensitivity to high temperature, stability toward endogenous digestive enzymes, and pH value. Thus, genes engineering for the development of enzymes with improved required properties is an area that has to be applied to fish feed. In enzyme processing, the stability of the enzymes should be ensured and maintained, and well-known methodologies, such as formulation or the application of post-spraying, can successfully address this issue. In addition, although the amount of enzyme added is small, it is necessary to further reduce the enzyme cost, to promote the industrial application of enzymes in aquatic feed under the current situation of fierce competition in the feed industry.

## Supplementary Information

Below is the link to the electronic supplementary material.

Supplementary file1 (DOCX 49 KB)

## Data Availability

Data sharing not applicable to this article as no datasets were generated or analysed during the current study.
