# Supplementary material for: Application of enzymes as a feed additive in aquaculture
Source: Mar Life Sci Technol. 2022 Apr 19;4(2):208–21. doi: 10.1007/s42995-022-00128-z (PMC10077164; doi:10.1007/s42995-022-00128-z)
Supplement: Supplementary file 1 — Supplementary file1 (DOCX 49 KB) [file 42995_2022_128_MOESM1_ESM.docx]

**Table S1**. Common enzymes used in aquaculture.

|  | **Biological effect** | **Targeted feedstuff** ^a^ | **Pretreatment process** | **Application effect** | **References** |
| --- | --- | --- | --- | --- | --- |
| Protease | Degradation of protein | Meat and bone meal | Pelleting | Enhance the growth and feed efficiency ratio | Lin et al. (2007) |
|  |  | Poultry byproduct meal | Pelleting | Improve nutrients retention (protein and lipid) | Shi et al. (2016) |
|  |  | Fish meal | Pelleting | Improve the growth, nutrient utilization, and retention of protein | Li et al. (2018) |
|  |  | Meat meal | Immobilization | Promote protein digestion and absorption | Tavano et al. (2018) |
|  |  | Cotton seed meal |  | Improve feed conversion ratio | Hassaan et al. (2019) |
|  |  | Soybean meal, and meat-and-bone meal | Microencapsulated | Improve growth, feed utilization and intestinal histology | Yao et al. (2019) |
|  |  | Spirulina, soybean meal, and fish meal |  | Improve the digestibility of Spirulina-based fish diets | Sharma et al. (2021) |
| Amylase | Degradation of starch | Soybean meal | Pelleting | Reduce waste emissions and environment pollution | Ogunkoya et al. (2006) |
|  |  | Fish meal | Pelleting | Improve feed utilization | Yildirim and Turan (2010) |
|  |  | Corn starch  meal | Pelleting | Increase dry matter digestibility and activity of endogenous enzymes | Kumar et al. (2016) |
|  |  | Fish meal and soybean meal | Microencapsulated | Improve the nutritional value | Guo et al. (2020) |
| Lipase | Degradation of fatty | Mixed meal | Pelleting | Improve feed conversion ratio | Ghomi et al. (2012) |
|  |  | Mixed meal |  | Increase body weight | Zamini et al. (2014) |
|  |  | Rapeseed meal |  | Improve intestinal growth and immunity | Liu et al., 2016) |
| Cellulase | Degradation of cellulose and non-starch polysaccharides | Peanut meal | Pelleting | Enhance specific growth rate and feed efficiency ratio | Ai et al. (2007) |
|  |  | Canola meal |  | No significant effects | Yigit and Olmez (2011) |
|  |  | Shredded duckweed and wheat flour meal |  | Enhance growth and intestinal flora | Zhou et al. (2013)  s |
|  |  | Corn starch meal | Pelleting | Improve the level of circulatory red blood cells | Adeoye et al. (2016) |
| Hemicellulases | Degradation of non-starch polysaccharides except cellulose | Mixed meal |  | Enhance carbohydrate utilization | Stone (2003) |
|  |  | Soybean meal and sunflower meal | Pelleting | Increase energy and digestibility of total carbohydrates | Mass et al. (2017) |
|  |  | Soybean meal | Pelleting | Improve weight gain and feed intake | Jacobsen et al. (2018) |
|  |  | Sunflower meal and wheat bran | Pelleting | Improve digestibility of non-starch polysaccharides, energy, and phosphorous and calcium | Mass et al. (2018) |
|  |  | Wheat gluten meal | Pelleting | Improve digestibility of dry matter, total carbohydrates, and non-starch polysaccharides | Mass et al. (2020) |
| Phytase | Degradation of phytate and phytic acid | Peanut meal | Pelleting | Increase retention of Phosphorus | Ai et al. (2007) |
|  |  | Corn starch meal | Pelleting | Increase weight growth rate | Adeoye et al. (2016) |
|  |  | Soybean meal, rapeseed meal and cottonseed meal | Pelleting | Improve the growth, nutrient utilization, and retention of Phosphorus | Li et al. (2018) |
|  |  | Wheat gluten meal | Pelleting | Improve nutrients digestibility | Maas et al. (2018) |
|  |  | Cotton seed meal | Pelleting | Improve apparent digestibility coefficient and retention of Phosphorus | Chen et al. (2019) |
|  |  | Mixture of Moringa seed meal and Moringa leaf meal | Pelleting | Improve nutrients and minerals absorption, reducing the nutrients leaching through feces | Shahzad et al. (2020) |
| Glucose oxidase | Catalyzes the dehydrogenation of β-D-glucose | Fish meal |  | Improve feed utilization and reduce feed wastage | Xie (2015) |
|  |  | Fish meal | Pelleting | Improve feed utilization and meat quality of fish | Wu (2016) |
|  |  | Fish meal |  | Improves absorption rate and palatability of feed | Huang (2017) |

^a^Data of targeted feedstuff were extracted from Table 19-1 in National Research Council. Nutrient Requirements of Fish and Shrimp. 2011. Washington, DC: The National Academies Press. https://doi.org/10.17226/13039.
